# Antimicrobial Photodynamic therapy enhanced by the peptide aurein 1.2

**DOI:** 10.1038/s41598-018-22687-x

**Published:** 2018-03-09

**Authors:** Laura Marise de Freitas, Esteban Nicolás Lorenzón, Norival Alves Santos-Filho, Lucas Henrique de Paula Zago, Marciana Pierina Uliana, Kleber Thiago de Oliveira, Eduardo Maffud Cilli, Carla Raquel Fontana

**Affiliations:** 1 Universidade Estadual Paulista (Unesp), Faculdade de Ciências Farmacêuticas, Araraquara, SP Rodovia Araraquara-Jaú, km 1, Campus Ville, CEP, 14800-903 Brazil; 2 Universidade Federal de Goiás, Instituto de Ciências Biológicas, Departamento de Bioquímica e Biologia Molecular, Campus II Samambaia, 74690-900 Goiânia, GO Brazil; 3 Universidade Estadual Paulista (Unesp), Instituto de Química, Araraquara, SP Rua Prof. Francisco Degni, 55, Quitandinha, CEP, 14800-060 Brazil; 4 Universidade Federal de São Carlos (UFSCar), Departamento de Química, Laboratório de Química Bioorgânica, Rodovia Washington Luis, km 235 - SP-310, São Carlos, SP CEP 13565-905 Brazil; 5 Present Address: Universidade Federal da Integração Latino-Americana (UNILA), Avenida Silvio Américo Sasdelli, 1842 - Vila A, Edifício Comercial Lorivo, CEP, 85866-000 Foz do Iguaçu, PR Brazil

## Abstract

In the past few years, the World Health Organization has been warning that the post-antibiotic era is an increasingly real threat. The rising and disseminated resistance to antibiotics made mandatory the search for new drugs and/or alternative therapies that are able to eliminate resistant microorganisms and impair the development of new forms of resistance. In this context, antimicrobial photodynamic therapy (aPDT) and helical cationic antimicrobial peptides (AMP) are highlighted for the treatment of localized infections. This study aimed to combine the AMP aurein 1.2 to aPDT using *Enterococcus faecalis* as a model strain. Our results demonstrate that the combination of aPDT with aurein 1.2 proved to be a feasible alternative capable of completely eliminating *E*. *faecalis* employing low concentrations of both PS and AMP, in comparison with the individual therapies. Aurein 1.2 is capable of enhancing the aPDT activity whenever mediated by methylene blue or chlorin-e6, but not by curcumin, revealing a PS-dependent mechanism. The combined treatment was also effective against different strains; noteworthy, it completely eliminated a vancomycin-resistant strain of *Enterococcus faecium*. Our results suggest that this combined protocol must be exploited for clinical applications in localized infections as an alternative to antibiotics.

## Introduction

According to data from the World Health Organization (WHO), infections caused by resistant bacteria affect more than 2 million people and cause more than 20 thousand deaths in the United States and the European Union annually^1^. Infection by resistant strains drastically reduces the probability of an effective treatment and elevates not only the morbidity and mortality of common infections but also increases the associated health costs, which reach billions per year^1,2^. In the past few years the WHO has been warning that the ‘post’-antibiotic” era, where common infections can kill again, is an increasingly real threat. And, even though the bacterial resistance continues to rise, the rate of new antibiotics development decreased in the past three decades, with no significant therapeutic class discovered since the 1980’s^1,3^.

The misuse and over-prescription of antibiotics have a huge role in the development of resistant strains. For example, oral infections, which are extremely common among the adult population, accounts for a great percentage of systemic antibiotics prescriptions, most of which could be avoided by using alternative practices^4^. Alternative antimicrobial treatments have been intensely pursued over the past decades, with focus on therapies that not only are able to eliminate resistant microorganisms, but also present characteristics that impair the development of new forms of resistance. Among the alternatives, antimicrobial Photodynamic Therapy (aPDT) has gained importance as an adjuvant to scaling and root planing in periodontal infections and to perform endodontic disinfections^5–7^, besides its application to other localized infections, especially from dermatological origin^8,9^.

The mechanism of aPDT relies on the uptake of a non-toxic dye named photosensitizer (PS) by the target cell and its activation by visible light of a specific wavelength in the presence of oxygen, originating highly damaging reactive oxygen species (ROS)^10^. Compared to conventional antimicrobial therapies, aPDT presents some advantages including the elimination of resistant microorganisms and secreted virulence factors, local delivery of PS, double selectivity (deleterious effect only on sites where both PS and light are delivered concomitantly), and immediate onset of action^8^. Due to the multiple and non-specific nature of death caused by ROS, and the short period of exposure to the PS, the expression of protective factors is hindered, making the development of resistance to aPDT by bacterial cells highly unlikely^11^. Although the local application of aPDT avoids systemic adverse effects, it usually requires high concentrations of photosensitizers and high-energy doses of light to completely eliminate infectious bacteria, particularly in the cases of persistent infections, which can lead to harm of host cells, impairing local immune response and tissue healing^8^, and even leading to tissue necrosis^12,13^.

To overcome such a drawback, aPDT can be combined with other agents or drugs, improving the overall result while reducing individual concentrations and avoiding host tissue damaging^14–16^. In particular, the combination of aPDT with antimicrobial peptides (AMP) has been investigated in the past few years, but, to the best of our knowledge, all studies involve the conjugation of the photosensitizer molecule with the AMP, the latter being employed as a targeting moiety^16^. Nevertheless, in addition to a possible loss of activity of both molecules due to the conformational changes caused by the conjugation, AMPs present an important antimicrobial activity by themselves to be used as a simple targeting.

The AMPs are naturally occurring oligopeptides (up to 50 amino acids residues) found both in prokaryotes and eukaryotes. They present a broad spectrum of action against microorganisms and compose the innate immune system of multicellular eukaryotes^17–19^. AMPs are, in their majority, α-helical amphipathic cationic molecules that have the cytoplasmic membrane as their main target^20–22^, a feature that confers them rapid killing effects, destroying bacterial cells within minutes once they reach their threshold concentration^23,24^. Moreover, they present prokaryotic selectivity as a result of their positive net charge, making them more attracted to the negatively charged bacterial cells, in comparison to mammalian cells^16,25^. An AMP of particular interest is aurein 1.2 (AU), the most studied one among the peptides found in the *Listeria spp*. Australian frogs^26^. Composed of 13-amino acid residues, aurein 1.2 have shown great potential to be used as an antimicrobial agent, presenting bactericidal activity against a wide variety of bacteria, and also presenting antiviral and anticancer activity^27^. In aqueous solutions, AU has no defined secondary structure, but it assumes a α-helical conformation once it encounters a hydrophobic environment, such as lipid membranes^28,29^.

Unlike antibiotics and similar to aPDT, AMPs do not act on specific cellular functions or pathways and have an immediate onset of action, leading to bacterial death within minutes^30,31^. Although the development of bacterial resistance to AMPs seems unlikely, some strains present mechanisms that can sense the presence of AMPs and modify the physical-chemical interactions between the cell membrane and the AMP, impairing their action^32^. Furthermore, high concentrations of the AMP (1 to 2 orders of magnitude higher than the threshold concentration for bacteria-killing) can destroy mammalian cells as well^24^, limiting the concentration range in which the AMP can be safely administered. In these scenarios, the combination of aPDT with AMPs, using the free non-conjugated molecules, shows potential as a safe alternative to conventional antimicrobials to treat localized infections.

Both aPDT and AMPs have demonstrated an enormous potential to treat bacterial infections, as highlighted by several studies^9,28,33–37^. However, so far, there are no studies combining the two approaches to obtain a synergistic effect and improved results without the need to administrate high concentrations of the drugs. In that sense, the aim of the present study was to combine the AMP aurein 1.2 to aPDT mediated by methylene blue, curcumin or chlorin-e6 to treat *Enterococcus faecalis*, an important nosocomial pathogenic species with high rates of antibiotic resistance. Our hypothesis was that aurein 1.2 could modify the membrane permeability and increase the PSs cellular uptake, thus improving the aPDT activity.

## Results

**Figure 1:**
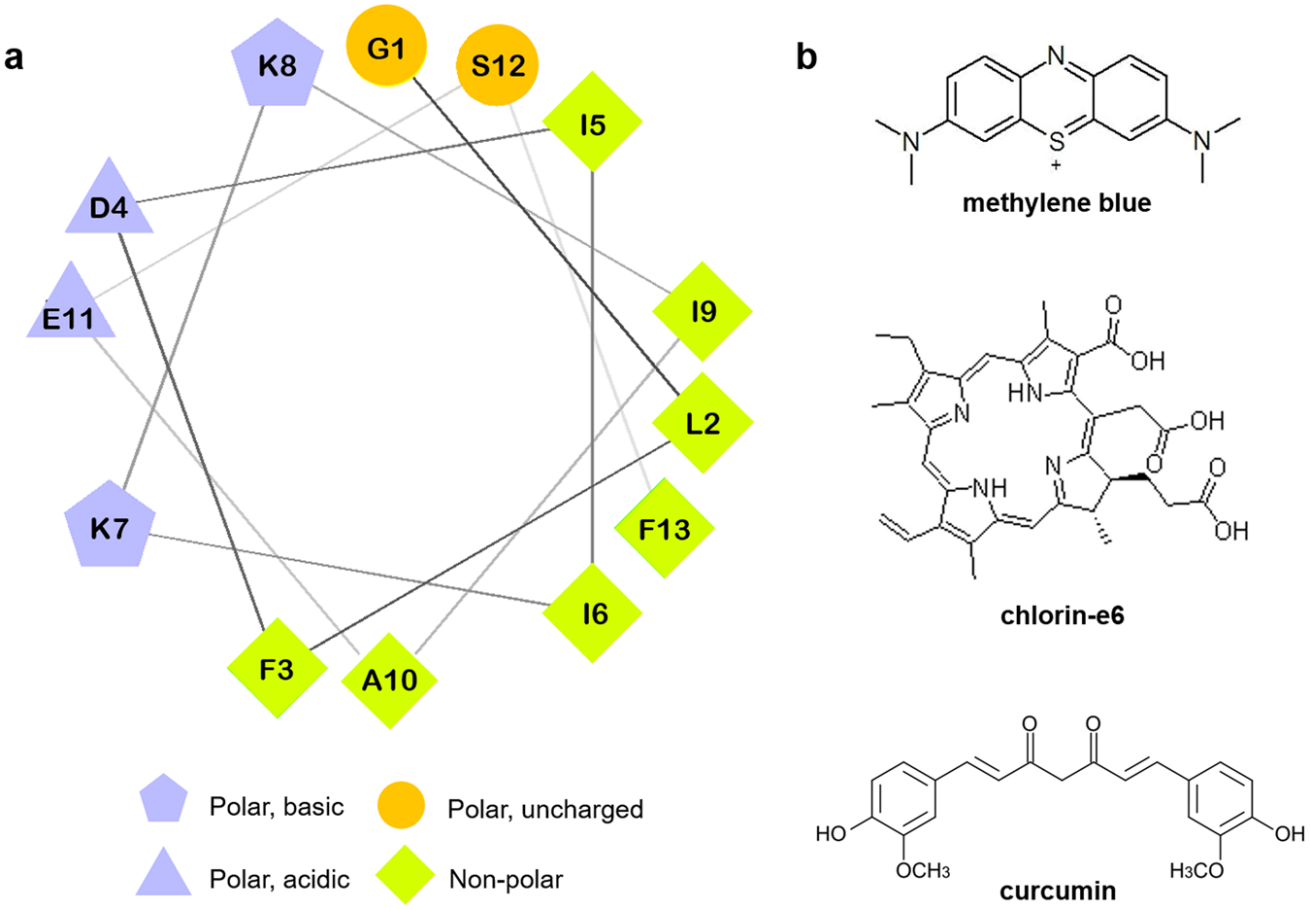
Aurein 1.2 and Photosensitizers structures. (**a**) Aurein 1.2 helical wheel diagram showing the disposition of each residue when the peptide assumes its α-helical conformation, evidencing the spatial “separation” of hydrophobic and hydrophilic residues, an important feature for the peptide mechanism of action. (**b**) Molecular structures of the photosensitizers methylene blue (top), chlorin-e6 (middle) and curcumin (bottom).

Figure 1a shows the Aurein 1.2 helical wheel diagram: this type of diagram demonstrate the disposition of the amino acids residues in an α-helical structure, revealing, in this case, the distribution of hydrophobic and hydrophilic residues on separate sides of the helix. The molecular structures of the photosensitizers are shown in Fig. 1b.

### Aurein 1.2 minimal bactericidal concentration (MBC)

**Figure 2:**
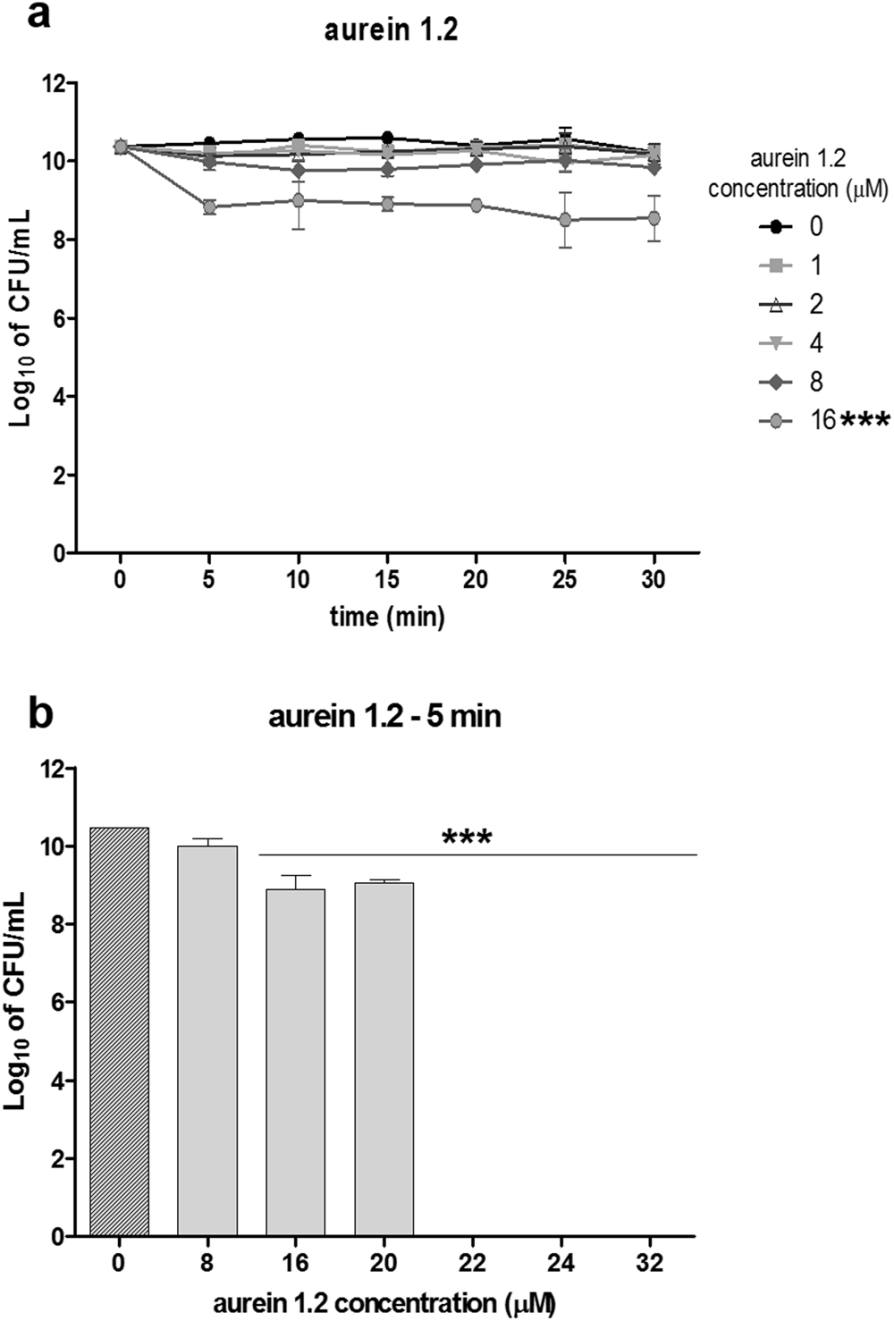
Aurein 1.2 minimal bactericidal concentration (MBC). Standardized suspensions of *Enterococcus faecalis* were submitted to incubation with aurein 1.2 for different time periods (**a**) and with different concentrations of the peptide for 5 minutes (**b**). The symbols and the columns represent the average of three independent assays (n = 9) and the bars represent the standard deviation. Two-way ANOVA (**a**) or One-way ANOVA (**b**); p < 0.0001. Asterisks indicate where there is a significant difference in comparison with the control (no aurein 1.2–0 µM).

To determine the activity of aurein 1.2 (AU), we first performed a time-kill assay to establish the ideal incubation time. We could observe in Fig. 2a that with up to 30 minutes of incubation there was no significant difference among the incubation times. We moved forward to determine the MBC using the 5-minute incubation, a standard pre-incubation time for most photosensitizers. Fig. 2b shows that AU MBC for *E*. *faecalis* was 22 µM, with an abrupt killing profile mimicking an all-or-nothing effect. These data are in agreement with a previous work^38^.

### Combination treatment (aPDT + AU)

For each photosensitizer used in this work, we carried out an extensive screening to determine the best combinations of energy dose and PS concentrations (data not shown). To combine aPDT with AU, we chose three concentrations of each PS combined with an energy dose that resulted in almost no bacterial reduction: 17, 34 and 68 µM of curcumin (CUR) irradiated with 12.5 J/cm² (average reduction of 5.2%); 21, 42 and 84 µM of chlorin-e6 (Ce6) irradiated with 30 J/cm² (average reduction of 10.3%); 39, 78 and 156 µM of methylene blue (MB) irradiated with 45 J/cm² (average reduction of 3.45%). As for AU concentration, two concentrations below the MBC were chosen, 8 and 16 µM, which resulted in approximately 16% and 32% of reduction, respectively.

**Figure 3:**
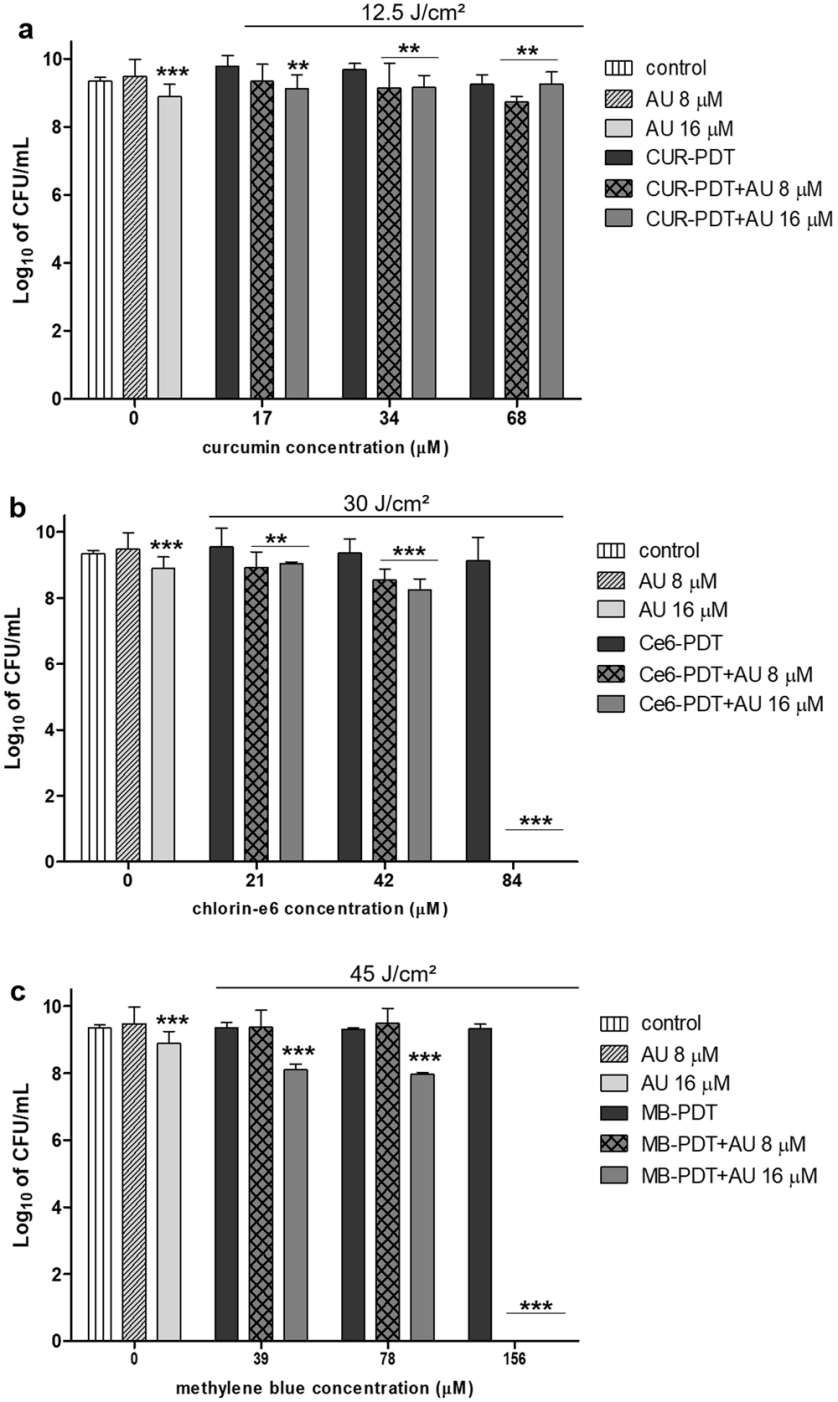
Combination treatment. Standardized suspensions of *Enterococcus faecalis* were submitted to sub-inhibitory photodynamic therapy combined or not to the peptide aurein 1.2. Columns represent the average of at least three independent assays performed in triplicates, and bars represent the standard deviation. The asterisks indicate where there is a significant difference in comparison with the control (no treatment). One-way ANOVA with Tukey’s post hoc. (*p < 0.05, **p < 0.01, ***p < 0.001). MB: methylene blue; Ce6: chlorin-e6; CUR: curcumin; AU: aurein 1.2.

The combination protocol was established incubating *E*. *faecalis* with both drugs simultaneously for 5 minutes and then irradiating the samples with the desired energy dose. Figure 3a shows combination treatment using CUR as PS. It is easily noted that combining CUR-PDT with AU improved to a little extent the bacterial reduction compared to CUR-PDT alone, but not compared to AU alone. This result indicates that the reduction observed for CUR-PDT + AU is mainly due to the AU action over the bacterium.

The combination of AU with Ce6-PDT and MB-PDT, however, improved bacterial reduction significantly, even leading to total bacterial elimination (over 10 log reduction, *i*.*e*. 100%), as seen in Fig. 3b,c respectively. For Ce6-PDT, the concentration of AU did not influence the results, with both 8 and 16 µM, but MB-PDT was more efficient when combined with 16 µM of AU for the lower MB concentrations (39 and 78 µM).

We also tested the combination protocol without light irradiation (Supplementary Figure S1), and no improved responses were observed, evidencing that the synergistic effect was dependent upon light activation (*i*.*e*., dependent on the photodynamic reaction).

### Photosensitizer uptake

**Figure 4:**
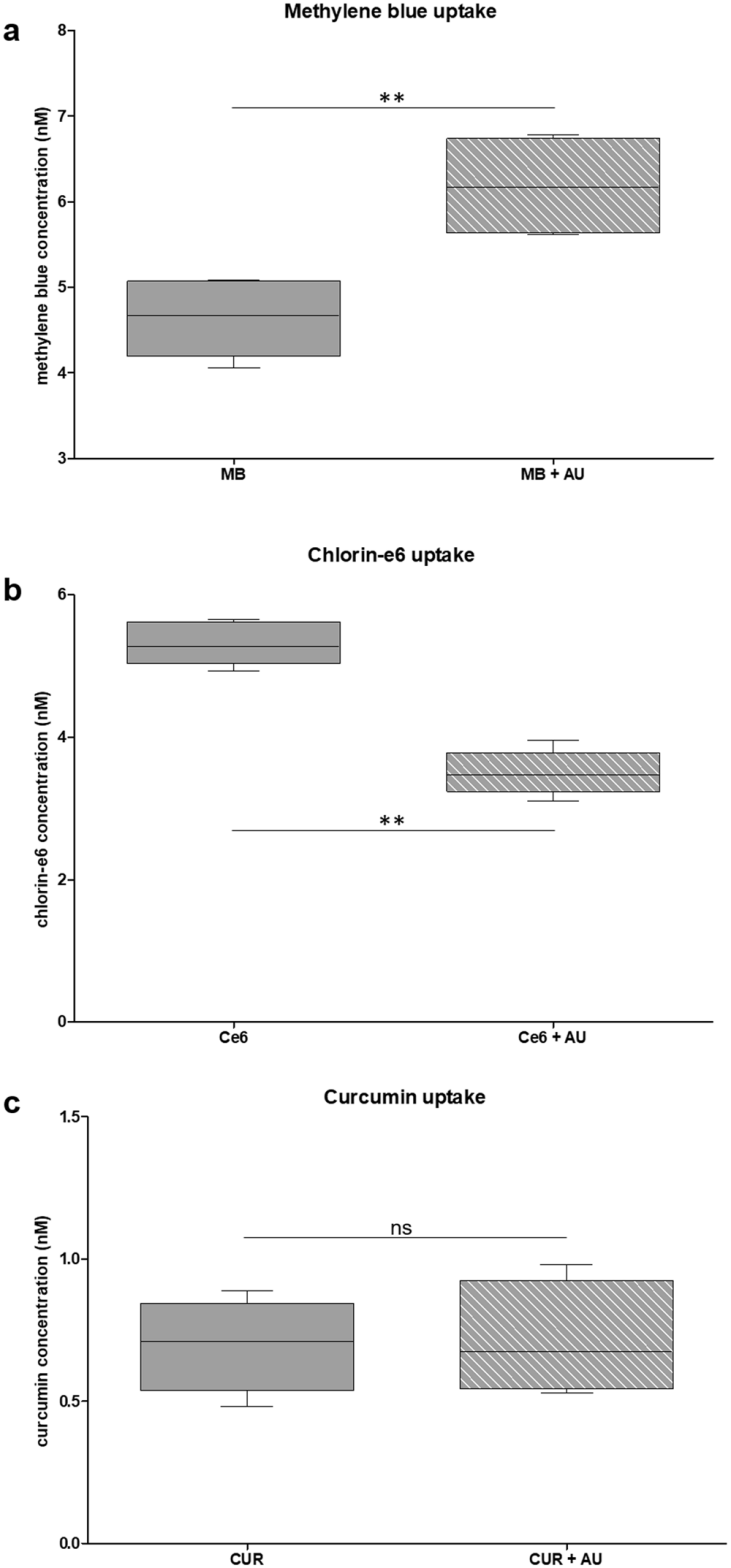
Photosensitizer uptake. Standardized suspensions of *Enterococcus faecalis* were incubated with methylene blue (156 µM; (**a**) chlorin-e6 (84 µM; (**b**) or curcumin (68 µM; (**c**) with or without aurein 1.2 (16 µM) for 5 minutes in the dark. The line inside the boxes represent the medians; boxes represent the minimum and maximum values, and whiskers represent the 10–90 percentiles. The y-axis represents the concentration of PS internalized by the totality of cells. Four independent assays (n = 12). The asterisk indicates where there is a significant difference between the groups (**p = 0.0022). Mann-Whitney test. MB: methylene blue; Ce6: chlorin-e6; CUR: curcumin; AU: aurein 1.2.

To evaluate the initial hypothesis, that AU could improve the PS uptake by bacterial cells by altering the membrane permeability, we sought to determine the amount of each PS taken by the cells in the absence and in the presence of AU, without light irradiation. By analysing Fig. 4a, is evident that the hypothesis was confirmed for MB-PDT: when AU is present in the media, bacterial cells uptake twice as much PS compared to the incubation with MB alone, which is most certainly the cause of the improved cell death response observed for this particular combination. In contrast, the presence of AU reduces the uptake of Ce6 (Fig. 4b), revealing not only that the hypothesis was not true for Ce6 but also that the synergistic effect can take place despite the PS uptake. The pattern of PS uptake was consistent for all concentrations of MB and Ce6 that were evaluated (see Supplementary Figure S2).

Although the statistical analysis did not reveal a significant difference between the two groups, there is a tendency of finding more CUR (Fig. 4c) inside the bacterial cells in the presence of AU, compared with the bacterium treated with the PS only. At the same time, the combination of AU with CUR-PDT did not work as expected. Therefore, this result indicates that the synergistic effect depends on the chemical structure of the PSs and their possible molecular interactions with AU macromolecule or by the simple increase of the PS concentration promoted by AU.

### ROS generation

**Figure 5:**
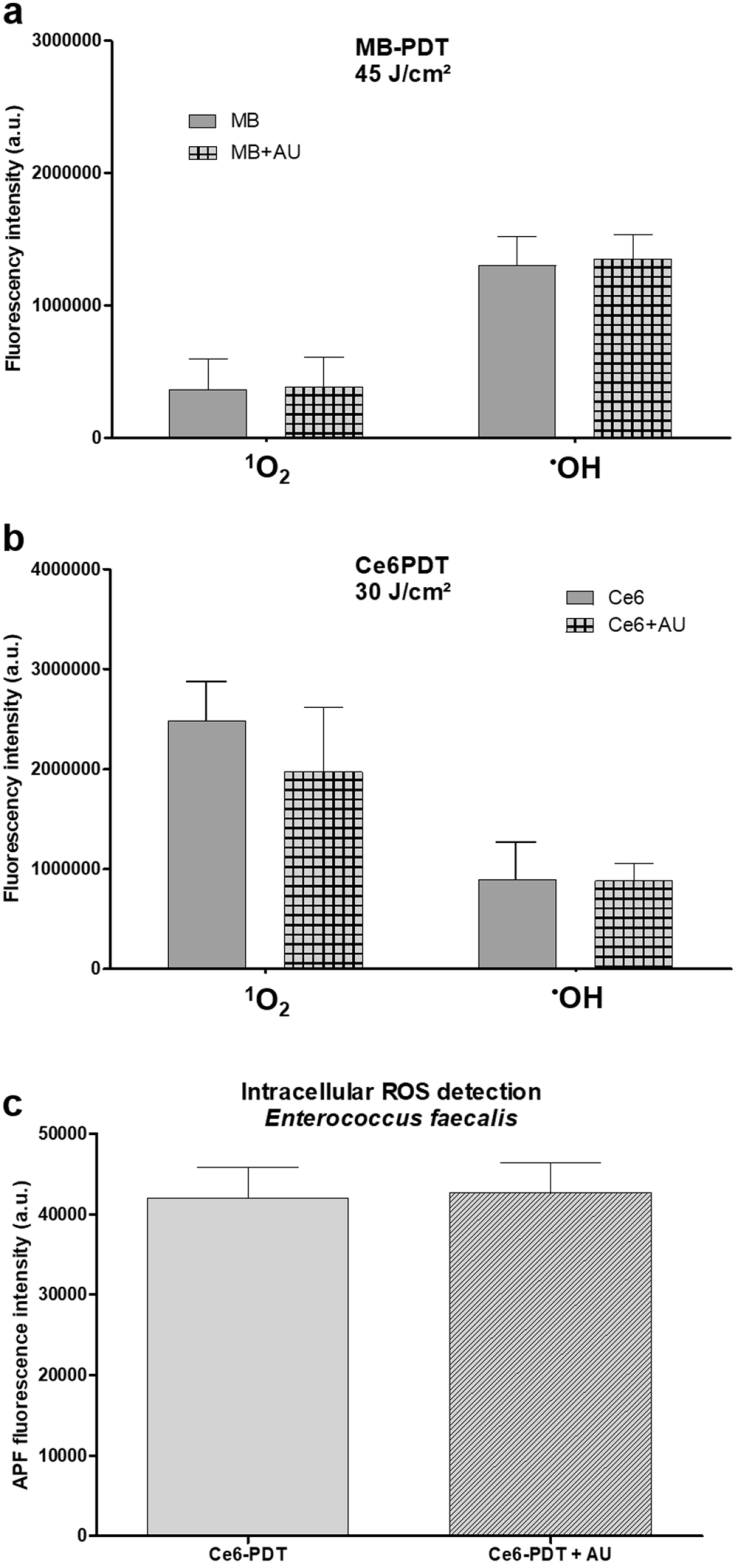
Reactive oxygen species (ROS) detection. Cell-free solutions of methylene blue (156 µM; (**a**) or chlorin-e6 (84 µM; (**b**) were combined or not with aurein 1.2 (16 µM) and incubated with ROS-detecting fluorescent probes to detect either hydroxyl radicals (^•^OH) or singlet oxygen (^1^O_2_) upon irradiation. (**c)** Standardized suspensions of *Enterococcus faecalis* were incubated with APF, washed, and then treated with Ce6-PDT or Ce6-PDT + AU. Statistical analysis (*t* test) revealed no significant difference between groups (p > 0.05 for all tests; n = 6). MB: methylene blue; Ce6: chlorin-e6; AU: aurein 1.2; a.u.: arbitrary unities.

For the two combinations that resulted in the synergistic effect, we conducted the ROS generation detection assay *in vitro* to evaluate if AU affects the photodynamic reaction. Solutions of MB or Ce6 were combined with ROS-detecting probes in the absence and in the presence of the peptide and irradiated with 45 (MB) or 30 (Ce6) J/cm². Generation of hydroxyl radical (^•^OH) and singlet oxygen (¹O_2_) were measured by indirect fluorescence and the results are illustrated in Fig. 5a,b.

Methylene blue is known to generate higher amounts of radical species (type I photodynamic mechanism) than singlet oxygen (type II mechanism) when employed in concentrations in the range employed in this study (156 µM)^4,9,39^. Therefore, the profile of generated ROS seen in Fig. 5a was expected. In addition, we observe that the presence of AU did not affect the photodynamic properties of MB, indicating once again that the synergistic effect obtained from the combination therapy is due to a higher intracellular concentration of the photosensitizer, increasing local generation of ROS.

Chlorin-e6, on the other hand, typically generates higher amounts of singlet oxygen than radical species, as seen in Fig. 5b and also described in the literature^40^. As observed for MB-PDT, the presence of AU also did not affect the photodynamic reaction of Ce6 to a significant matter. The synergistic effect, therefore, is not due to a higher bulk generation of ROS. Nevertheless, since the chlorin-e6 results were not a consequence of higher PS uptake, we investigated if the combined protocol could be increasing the ROS generation within the bacteria. Fig. 5c shows, however, that the presence of the peptide does not interfere in any way with the photodynamic reaction, pointing to a synergistic mechanism based on direct molecular interactions.

### Photobleaching

**Figure 6:**
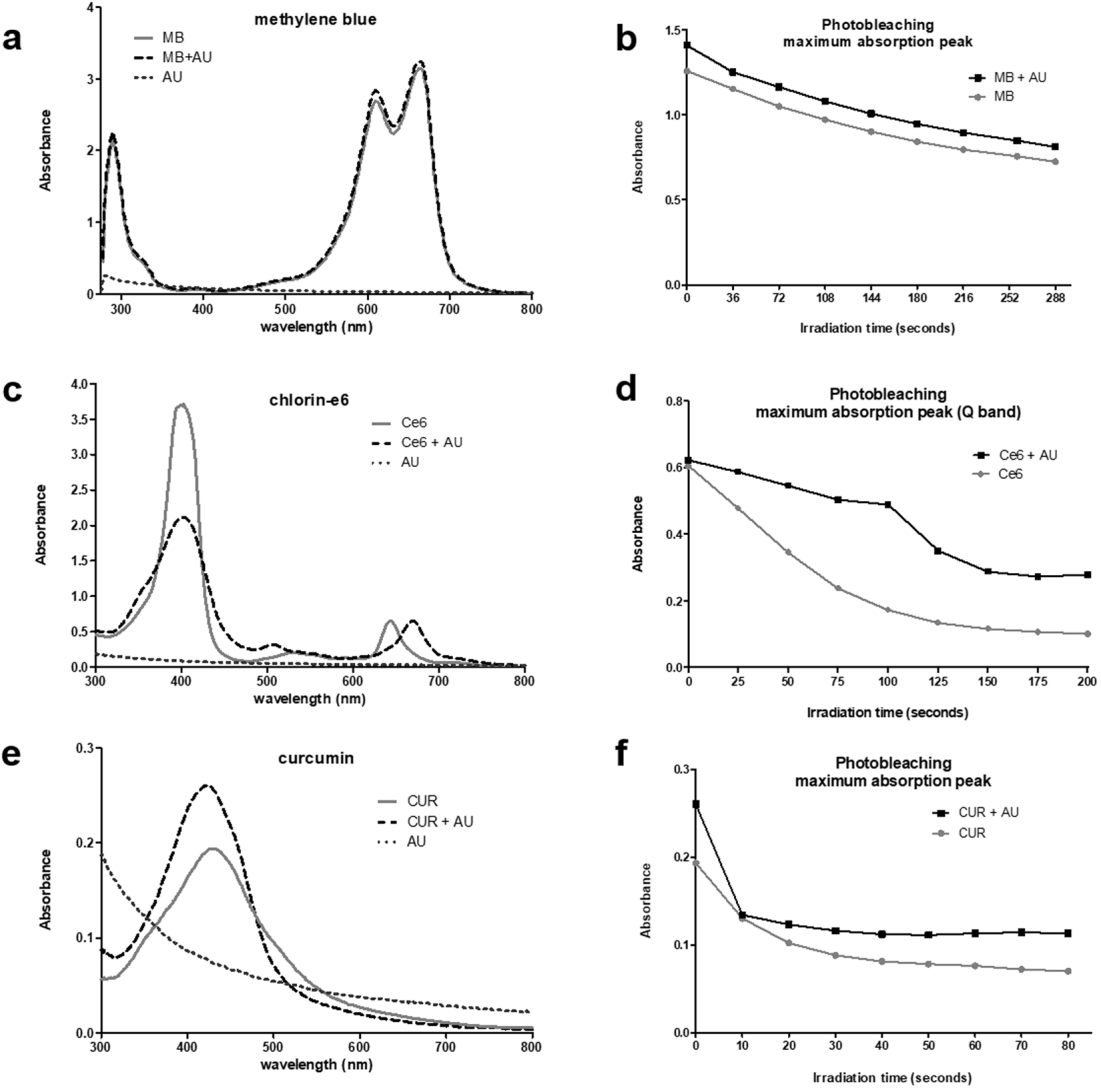
Absorbance spectrum and photobleaching of photosensitizers. (**a**) Normalized UV-VIS spectra of MB (; 156 µM), AU (; 16 µM) and MB combined with AU (), in aqueous solutions. (**b**) Photobleaching kinetics assessment of MB alone and in the presence of AU irradiated with a total energy dose of 45 J/cm². Statistical analyses revealed no significant difference between the two slopes (p = 0.2581). (**c**) Normalized UV-VIS spectra of Ce6 (; 84 µM), AU (; 16 µM) and Ce6 combined with AU (), in aqueous solutions. (**d**) Photobleaching kinetics assessment of Ce6 alone and in the presence of AU irradiated with a total energy dose of 30 J/cm². Statistical analyses revealed a significant difference between the two slopes (p < 0.0001). (**e**) Normalized UV-VIS spectra of CUR (; 68 µM), AU (; 16 µM) and CUR combined with AU (), in aqueous solutions. (**f**) Photobleaching kinetics assessment of CUR alone and in the presence of AU irradiated with a total energy dose of 12.5 J/cm². Statistical analyses revealed a significant difference between the two slopes (p = 0.0244). All assays were executed with Synergy H1M (Synergy H1 Multi-Mode Reader, BioTek, Winooski, VT, USA). CUR: curcumin; Ce6: chlorin-e6; MB: methylene blue; AU: aurein 1.2.

We evaluated the absorption spectra of each PS when combined with AU to investigate any alterations in the PS’s optical properties, as well as their photobleaching profile. Methylene blue spectrum (Fig. 6a) did not suffer any shifts or intensity variations when AU was present, neither did its degradation rate upon light irradiation (Fig. 6b). These results indicate that the peptide and MB do not present significant chemical or physical interactions, which is evidenced by the UV-Vis analysis.

On the other hand, Ce6 spectrum recorded in the presence of AU presented an eye-catching alteration (Fig. 6c): the peptide reduced the intensity of the Soret band (400 nm) in approximately 80% due to a broadening of the peak and promoted a red-shift on the more intense Q-band (from 652 to 665 nm). These alterations indicate the formation of Ce6 aggregates and/or Ce6-AU complexes^41^. In addition, it is important to highlight that the peptide seems to have a protective effect over the Ce6 photobleaching, reducing the degradation rate (Fig. 6d).

In an opposite way, the curcumin spectrum in the presence of AU (Fig. 6e) exhibited alterations compatible with the CUR-AU complexes: the AU presence led to a narrower maximum peak with an increased absorption intensity and a blue-shift (from 428 to 418 nm). AU is an amphipathic molecule with surfactant features, capable of interacting with both the highly hydrophobic CUR and the water, forming a stable and soluble complex. This characteristic could help improving curcumin solubility at the same time it diminishes the PS availability in solution, even retarding its photobleaching (Fig. 6f).

### Circular Dichroism

**Figure 7:**
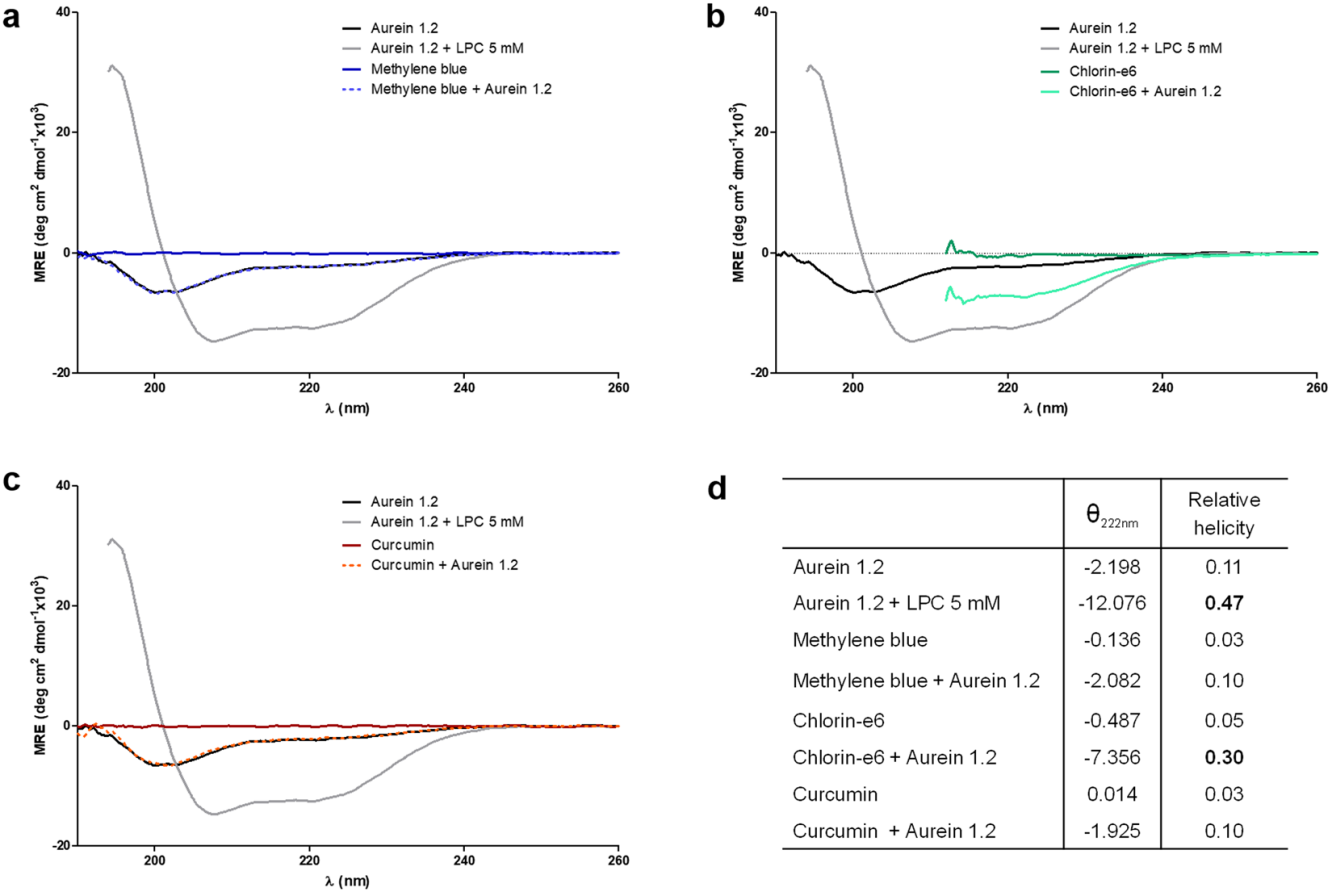
Circular dichroism of the AU. CD spectra of AU (16 µM) were recorded in aqueous solution (phosphate buffer 0.1 M pH 7.2) prior and after the addition of LPC (5 mM), MB (156 µM; (**a**), Ce6 (84 µM; (**b**), or CUR (68 µM; (**c**). CDs are an average of ten recorded spectra converted to mean-residue ellipticity [θ] (in deg cm^2^ dmol^−1^×10^3^) for each sample. (**d**) Mean residue ellipticity and helicity relative to Aurein 1.2 in different environments. CUR: curcumin; Ce6: chlorin-e6; MB: methylene blue; AU: aurein 1.2; LPC: lysophosphatidylcholine.

After investigating AU’s effect on the PS properties, we evaluated the PS’s effect over AU by analysing the peptide conformation in the presence of each PS, as shown in Fig. 7. AU in aqueous solution displayed typical spectra for disordered structures, with the induction of a α-helical structure occurring after the addition of the LPC micelles (Fig. 7a, black and grey lines), evidenced by two negative bands at 208 and 222 nm, as previously described^26^. The addition of solutions of MB or CUR to AU did not interfere with the peptide conformation (Figs. 7a,c, light and dark blue lines, and light and dark orange lines, respectively). When a Ce6 solution was added, however, a α-helical pattern was recorded (Fig. 7b, light and dark green lines), indicating that Ce6 provides a non-polar environment capable of inducing AU structuration, similar to that induced by LPC, as evidenced by the relative helicity described in Fig. 7d.

### Chlorin-e6 and aurein 1.2 effects over the cell membrane

In light of the results presented so far, we hypothesized that the mechanism of the synergistic effect between Ce6-PDT and AU could be based on an increased instability of the bacterial membrane due to the accumulation of Ce6-Ce6 aggregates and/or Ce6-AU complexes. We investigated that hypothesis by means of zeta potential analysis and propidium iodide (PI) uptake, which would provide us with information regarding the size and net charge of the cell, and the membrane permeability, respectively.

**Figure 8:**
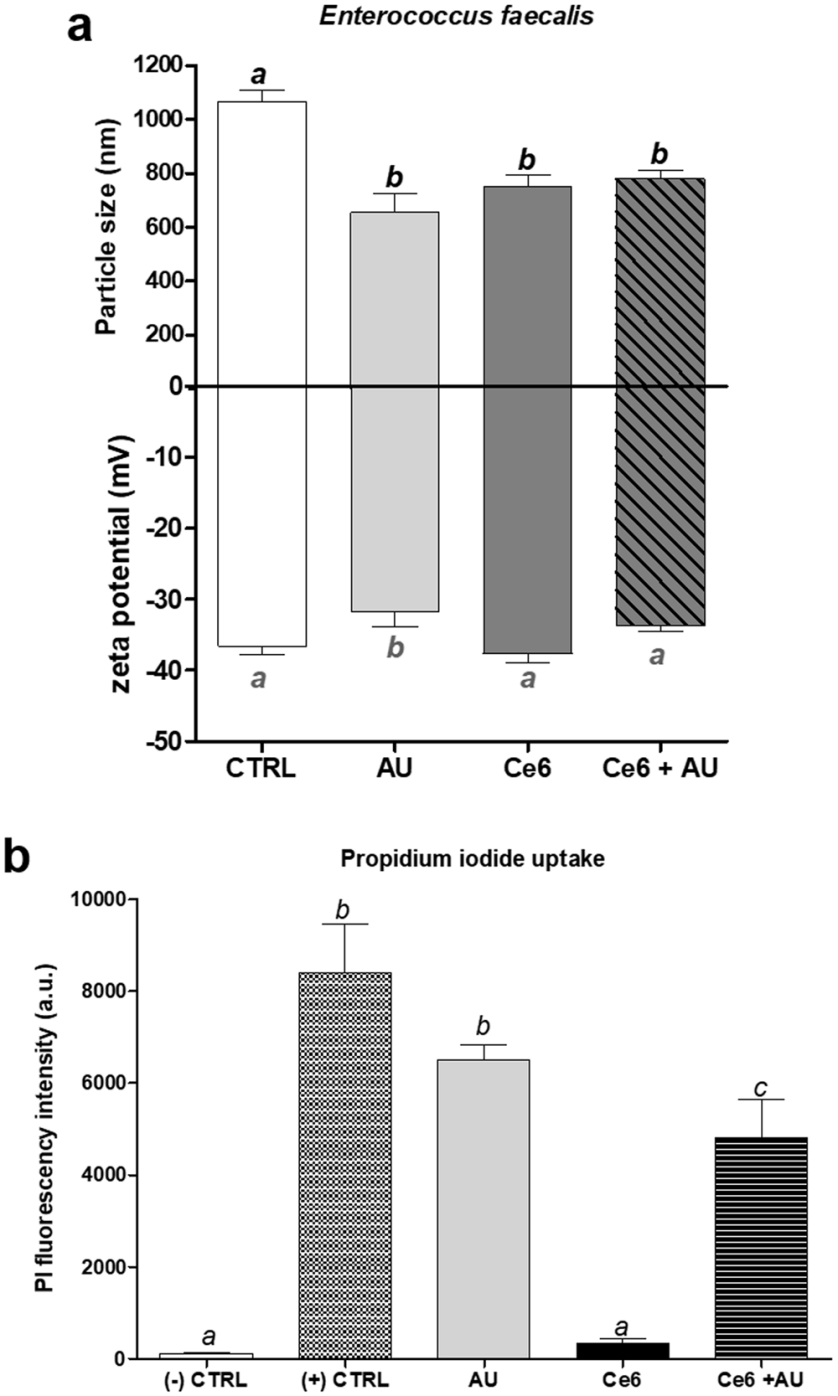
Chlorin-e6 and aurein 1.2 effects over the cell wall. (**a**) zeta potential: Standardized suspensions of *Enterococcus faecalis* were incubated with AU (16 µM), Ce6 (84 µM) or Ce6 + AU for 5 minutes in the dark. Particle size and the zeta potential of the bacterial cells were determined for each treatment and compared with the control (no treatment). The columns represent the average of 30 readings from three independent assays, bars represent the standard deviation. Different letters indicate a significant difference between groups. One-way ANOVA with Tukey’s *posthoc*. Particle size: p < 0.0001; zeta potential: p = 0.0282. **(b)** propidium iodide uptake: Standardized suspensions of *Enterococcus faecalis* were incubated with isopropanol 70%, AU (16 µM), Ce6 (84 µM) or Ce6 + AU for 5 minutes in the dark. Cells were washed to remove treatment and incubated with PI for 5 min. After removing excess PI, fluorescence was read at 535/617 nm. The columns represent the average of three independent assays performed in triplicates, bars represent the standard deviation. Different letters indicate a significant difference between groups. One-way ANOVA with Tukey’s *posthoc*, p < 0.0001. (−) CTRL: negative control (no treatment); (+) CTRL: positive control (isopropanol 70%); Ce6: chlorin-e6; AU: aurein 1.2.

Figure 8a shows that AU induced both an alteration on the particle size and on the zeta potential of *E*. *faecalis*, as expected: the positive charges of the peptide neutralize some of the negative charges of the bacteria. The reduced cell size is most likely due to cell permeabilization, as seen in Fig. 8b: the treatment with AU increases the uptake of PI, a DNA-binding probe that is impermeant to the intact cell membrane. The treatment with Ce6, on the other hand, did not affect the zeta potential, since it is a neutral molecule, but also had an effect over the particle size (Fig. 8a), even though the cell permeabilization was not significantly altered (Fig. 8b). By combining the two molecules, however, we observed the same reduction on the particle size, no significant alteration on the zeta potential (Fig. 8a), and cell permeabilization 26% lower than the permeabilization caused by AU alone (Fig. 8b), meaning that AU was not fully available to bind to the membrane. Taken together, the results confirm the formation of Ce6-AU complexes. In addition, even though the cell permeabilization is reduced in the presence of the complexes, cell death is enhanced, pointing toward a higher membrane instability due to the accumulation of those complexes.

### Activity against other clinically relevant pathogens

**Figure 9:**
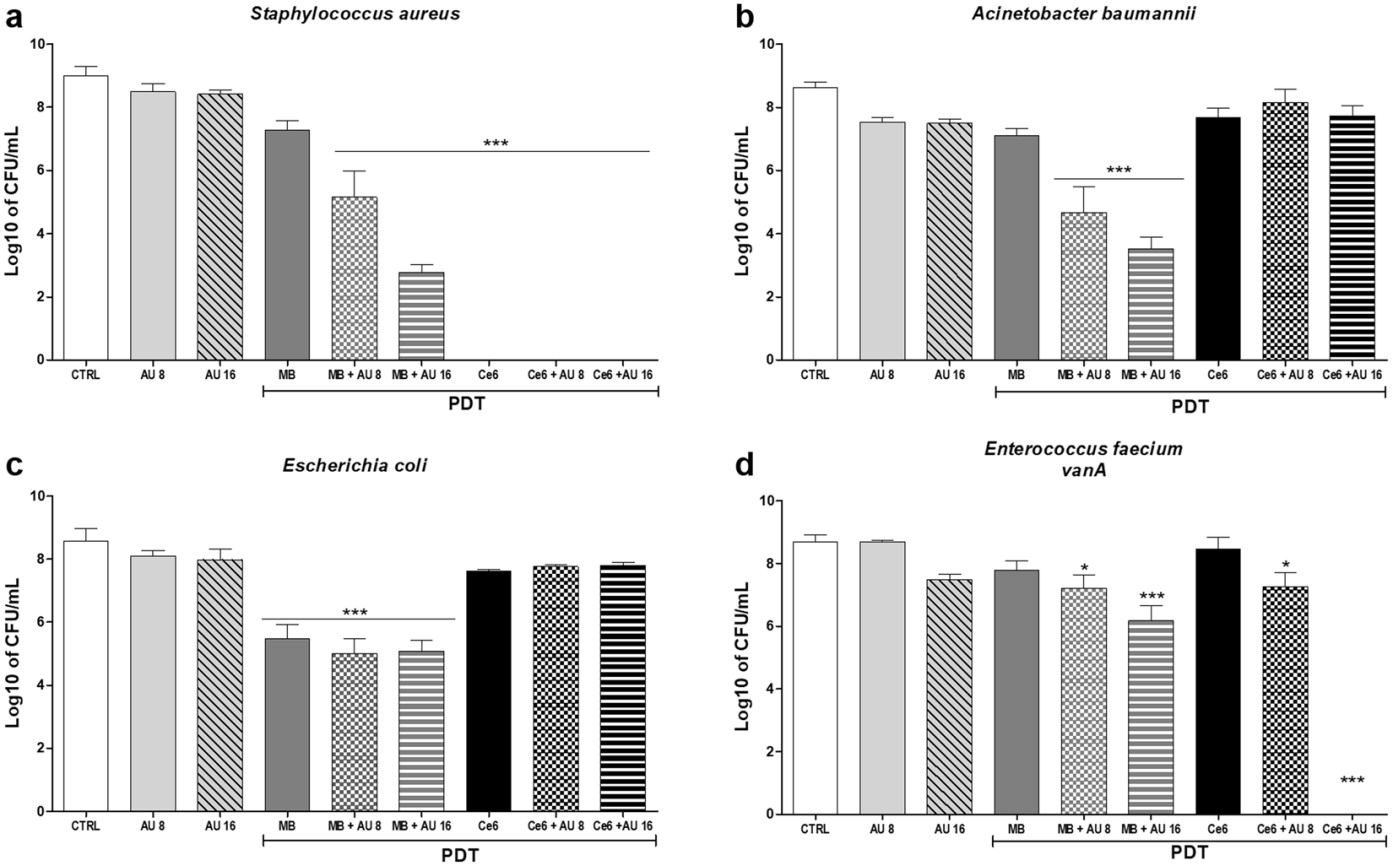
Combined treatment against clinically relevant strains. Standardized suspensions of *Staphylococcus aureus* (**a**), *Acinetobacter baumannii* (**b**), *Escherichia coli* (**c**) or *Enterococcus faecium VRE* (**d**) were submitted to photodynamic therapy combined or not to the peptide aurein 1.2. MB-PDT: 156 µM of MB + 45 J/cm²; Ce6-PDT: 84 µM of Ce6 + 30 J/cm². Columns represent the average of at least three independent assays performed in triplicates, and bars represent the standard deviation. The asterisks indicate where there is a significant difference in comparison with the control (no treatment). One-way ANOVA with Tukey’s post hoc. (*p < 0.05, **p < 0.01, ***p < 0.001). MB: methylene blue; Ce6: chlorin-e6; CUR: curcumin; AU: aurein 1.2.

Once the synergistic effect was established, and using the same parameters that eliminated the totality of *E*. *faecalis*, we tested the combination treatment against other clinically important species: *Staphylococcus aureus* (gram-positive), *Acinetobacter baumannii* (gram-negative), *Escherichia coli* (gram-negative) and *Enterococcus faecium VRE (vanA;* gram-positive). As it can be seen in Fig. 9, all four strains had similar sensitivities to the peptide, which was not statistically different from the controls. MB-PDT combined with AU at the higher concentration (16 µM) reduced the bacterial load of *S*. *aureus* and *A*. *baumannii* in 6 and 5 log10 (99.9999 and 99.999%), respectively, but reduced *E*. *faecium VRE* in only 2.5 log10 (~99%) and had no significant improvement in the reduction of *E*. *coli* in comparison with MB-PDT alone. The two gram-negative strains were refractory to Ce6-PDT, while *S*. *aureus* exhibited great sensitivity, being eliminated even with the Ce6-PDT alone, and *E*. *faecium* was successfully eliminated by combining Ce6-PDT with AU at 16 µM. This is an important result, given that *E*. *faecium VRE* is one of the most challenging strains in the clinical practice.

## Discussion

Considering the limitations of this *in vitro* study, the combination of aPDT with the AMP aurein 1.2 was proved highly effective in eliminating *Enterococcus faecalis*, one of the most clinically relevant bacterial strains nowadays, capable of surviving in hostile environments and resistant to antimicrobial agents, especially vancomycin^42^. When the PSs employed in the combination protocol were methylene blue or chlorin-e6 a synergistic effect was observed, but not when employing curcumin. The photodynamic reaction (ROS generation) was found to be the limiting factor of the synergistic effect. PS-uptake and ROS generation assays revealed that the synergism obtained from the combination of MB-PDT with AU is most probably due to a higher internalization of MB by bacterial cells as a consequence of an increase in the membrane permeability provoked by AU, in accordance with our initial hypothesis. The higher internalization of MB increases the production of ROS inside the cells, amplifying the photodynamic effect.

On the other hand, for Ce6 our results indicate that the addition of AU induces the formation of Ce6-AU complexes without affecting its ROS-generation properties. Ce6 is protected from photobleaching when AU is present, which could help prolong the half-life of the PS, therefore favouring the global photodynamic effect. In addition, the Ce6-AU complexes seem to hamper the PS molecule internalization, probably causing them to accumulate on the plasmatic membrane, leading to a higher instability of this structure and making it easier to break down upon light activation. This inference is based on the principle that the binding of molecules to a lipid bilayer alters its physical properties, changing both its thickness and elasticity^43^, and is in accordance with the data obtained by Vermathen *et al*.^44^, whose study demonstrate that the higher the degree of aggregation of Ce6, the lower its internalization, and the data from Fuchs *et al*.^45^, which demonstrate that higher membrane instability makes the cells more susceptible to aPDT. Moreover, Ce6 is not significantly ionized at neutral pH (pKa of its carboxylic groups ranges between 7 and 8^44^), and, therefore, is not suitable for an ionic interaction with the cationic peptide: the interaction between the two molecules is most likely of hydrophobic nature, as demonstrated by previous studies involving the interaction of peptides with porphyrins^46,47^, but dipole-dipole interactions and hydrogen bonds are also possible. That could explain how Ce6 was able to induce a premature α-helix conformation in AU, which could have favoured the peptide interaction with the membrane^29,48^. Finally, Ce6 being a neutral PS, the positive charges of the peptide present in the Ce6-AU complexes favours Ce6 binding to the bacterial cell wall.

Combining AU with CUR-PDT had no improvement over the results, prevailing only the AMP activity. Analysing the absorption spectra of curcumin with and without AU, the reason for the absent synergistic effect seems to be the formation of a soluble and stable complex, protecting CUR from photobleaching. Face-to-face curcumin conjugates scatter light diminishing the absorption intensity^49^; the amphipathic nature of AU could be dismantling those conjugates, justifying the narrower and more intense absorption peak after the addition of the peptide. The improved curcumin solubility is probably due to the intermolecular hydrogen bonds formation, as observed in the interaction of CUR and cationic surfactants^49,50^. This effect was expected to improve CUR activity as a photosensitizer since it would become more available in solution; however, the interaction seems to take place in such a way that impairs the photodynamic effect, most likely by decreasing CUR reactivity in solution. Moreover, the fact that the photodynamic reaction was determined as the limiting factor for the synergistic effect, and CUR has the lowest ROS yield of the three PS, helps understand why this natural PS did not have a synergistic action with the peptide.

The combination protocol exploited in this study has also been proven to be effective against other clinically relevant strains commonly identified as causes of life-threatening nosocomial infections, related to high levels of antibiotic resistance. Using the same parameters that resulted in complete killing for *E*. *faecalis*, MB-PDT combined with AU had synergistic effects over all strains, except *E*. *coli*, with no significant improvement over cell killing in comparison with MB-PDT alone. That is because *E*. *coli* is particularly less sensitive to aurein 1.2, as demonstrated in previous studies^51,52^, and therefore the presence of AU could not increase the penetration of MB. The two gram-negative strains were refractory to Ce6, which prevented a synergism from taking place. That is related to their cell wall structure: gram-negative strains are less susceptible to neutral (such as Ce6) and anionic photosensitizers, but susceptible to cationic ones (such as MB)^8^. Finally, *S*. *aureus* was extensively susceptible to Ce6-PDT, even without AU, as previously reported^53^, and *E*. *faecium* VRE was successfully eliminated by the combination protocol, an unprecedented achievement. In summary, MB-PDT combined with AU showed a broader spectrum of activity against clinically important pathogens, but Ce6-PDT + AU had a more dramatic effect over the total elimination of the highly tolerant *E*. *faecium* and *E*. *faecalis*.

Taken together, the results obtained in this study reveal that combining aPDT with an AMP can improve bacterial killing with minimum concentrations of both PS and AMP and low light doses, which in turn would minimize adverse effects to host tissue. As stated above, resistance to aPDT has been proven unlikely; resistance to AMP, although possible, would be practically abolished by employing the peptide in low concentrations in the combination protocol. This synergistic approach has the potential to eliminate localized infections and, at the same time, minimize the use of systemic antimicrobials and prevent the development of new resistance profiles.

## Conclusion

The combination of aPDT with the AMP aurein 1.2 proved to be a feasible alternative to conventional antimicrobial agents to potentially overcome bacterial resistance, capable of eliminating two of the most resistant strains responsible for community and nosocomial infections, *E*. *faecalis* and *E*. *faecium*, and significantly reduce the viability of *S*. *aureus*, *A*. *baumannii* and *E*. *coli*. Within the tested conditions, aurein 1.2 is capable of potentiating aPDT mediated by methylene blue or chlorin-e6, but not by curcumin, evidencing a photosensitizer-dependent synergistic effect. The mechanisms of the synergism, in fact, seems to be related to the PS molecule: even though we observed a similar synergistic effect using both MB and Ce6 in the presence of the peptide, MB accumulated on the inside, while Ce6 accumulated on the outside of the bacterial cell, not only revealing different mechanisms of action, but also evidencing the multiple-target nature of the photodynamic effect. Our results highlight the importance of the evaluation of several combinations when seeking for a synergistic effect and suggest that combination protocols employing alternative approaches must be exploited for clinical applications in localized infections.

## Material and Methods

### Bacterial strains and culture conditions

The model strain used in this study was *Enterococcus faecalis* (ATCC® 29212™), obtained from the National Institute of Quality Control in Health (INCQS) from the Oswaldo Cruz Foundation (FIOCRUZ - Manguinhos, RJ, Brazil). *E*. *faecalis* was cultured in blood agar (defibrinated sheep blood 5%; BHI 2,6%; TSA 2%; yeast extract 1%) at 35 °C.

Other strains used were: *Staphylococcus aureus* (ATCC® 25923™), *Acinetobacter baumannii* (ATCC® 19606™), *Escherichia coli* (ATCC® 25922™) and *Enterococcus faecium VRE* (ATCC® 700221™), all kindly provided by Dr. Nilton Lincopan (Biomedical Sciences Institute of the University of Sao Paulo). *S*. *aureus*, *A*. *baumannii* and *E*. *coli* were cultured on BHI agar, and *E*. *faecium* was cultured on blood agar; all cultures were kept at 35 °C in aerobic conditions.

### Photosensitizers

This study compared three different photosensitizers: a phenothiazine derivative (methylene blue); a chlorophyll derivative (chlorin-e6); and a turmeric curcuminoid (curcumin).

Methylene blue was purchased from Sigma (Sigma-Aldrich Co. LLC, St. Louis, MO, USA). Chlorin-e6 was synthesized as described by Uliana and co-workers^53^, and curcumin was synthesized as described by Wichitnithad and co-workers^54^.

### Light sources

Curcumin was activated by light at 450 nm (155 mW/cm²); methylene blue and chlorin-e6 by light at 660 nm (151 mW/cm²). Both light sources consisted of 48 LEDs with variable irradiances assembled as a compact illumination system with a homogeneous illumination area and a cooling device (IrradLED® – biopdi, Sao Carlos, SP, Brazil). The power density of the incident radiation was measured using a power meter (Coherent®, Santa Clara, CA, USA).

### Aurein 1.2 (AU) synthesis

AU synthesis (GLFDIIKKIAESF-NH_2_) was performed manually by solid phase peptide synthesis (SPPS) using the standard Fmoc (9-fluorenylmethyloxycarbonyl) protocols on a Rink MBHA resin (0.6 mmol/g), as described by Lorenzón and co-workers, 2013^26^. The peptide synthesis and structure were confirmed by HPLC and mass spectroscopy (See Supplementary Figure S4).

### Photodynamic Therapy and Treatment with aurein 1.2

Photosensitizers (PS) or AU solutions were prepared at twice the desired working concentration and 50 µL added to the correspondent wells of 96 wells plates, in triplicates. The bacterial strains, grown in agar plates, were scraped from the agar and suspended in 3 mL of BHI broth at a concentration of ~5 × 10^9^ cell/mL, confirmed by spectrophotometry reading. Aliquots of 50 µL of the inoculum were transferred to each well of the plate, yielding a final volume of 100 µL, and the dilution of both solution and inoculum by 50%, reaching the work concentrations. Growth control was composed of 50 µL of inoculum and 50 µL of BHI broth (final bacterial “concentration” = ~1 × 10^9^ cells), incubated for the same period as the treated groups. For aPDT, the bacteria was incubated for 5 minutes in the dark and then irradiated for the appropriate time to yield the desired energy densities (J/cm²); irradiation was conducted in a switched way (60 s LED on, 60 s LED off), from underneath. For AU treatment, *E*. *faecalis* was incubated for 5, 10, 15, 20, 25 or 30 minutes in the dark. After incubations, bacterial suspensions were serially diluted and plated (5 µL of each dilution, as a spot). Colonies were counted after 1–2 days of incubation. Each assay was performed in triplicate and in at least 3 different occasions. For the combined therapy, bacterial suspensions were incubated with the PS and AU simultaneously for 5 minutes in the dark and then irradiated at appropriated wavelength and energy dose. All other steps were the same as described above. Treatments of *S*. *aureus*, *A*. *baumannii*, *E*. *coli* and *E*. *faecium* followed the same protocol; conditions were the same ones that resulted in total elimination of the bacterial load of *E*. *faecalis*.

### Photosensitizer uptake assay

This protocol was modified from Tegos & Hamblin^55^. Bacterial suspensions (10^8^ cells/mL) were incubated in BHI broth at room temperature in the dark for 5 min with the photosensitizer (PS), aurein 1.2, or PS + aurein 1.2 in the same concentrations as were used for the synergistic aPDT experiments. A suspension incubated with BHI only was used as a control. All incubations were carried out in triplicates. Cell suspensions were centrifuged (6500 rpm for 5 min), the supernatant was discarded, and bacteria were washed twice with 1 mL of sterile phosphate buffer (0.1 M, pH 7.4) and centrifuged again (6500 rpm for 5 min). At last, the pellet was dissolved in 2 mL of 0.1 M NaOH-1% sodium dodecyl sulfate (SDS) for 24 h for digestion, conferring a cell extract as a homogenous solution. The extracts had their fluorescence measured with a spectrofluorometer in the endpoint reading mode (Synergy H1 Multi-Mode Reader, BioTek, Winooski, VT, USA). For methylene blue, the excitation wavelength was 660 nm and the emission was 690 nm; for chlorin-e6, the excitation wavelength was 500 nm and the emission was 670 nm; for curcumin, the excitation wavelength was 450 nm and the emission was 640 nm. PS dissolved in NaOH-SDS in several concentrations were used to make calibration curves, which were used for determination of PS concentration in the extract.

### Photobleaching

UV-VIS spectra were determined before, during, and after light irradiation of PSs in the absence and presence of AU. All solutions were prepared using the same concentrations that resulted in a synergistic effect (or not, in the case of curcumin) in the combination therapy assay (AU 16 µM; Ce6 84 µM; CUR 68 µM; MB 156 µM). Total light dose (also derived from the combination therapy assay: 30 J/cm² for Ce6; 12.5 J/cm² for CUR; and 45 J/cm² for MB) was divided into 8 irradiations of 10, 25 or 36 seconds each, according to the PS. Maximum absorption peaks at each time were recorded. Measures were obtained with Synergy H1M (Synergy H1 Multi-Mode Reader, BioTek, Winooski, VT, USA).

### ROS detection

#### Cell-free system (solution)

ROS-detecting fluorescent probes 3′-p-(aminophenyl) fluorescein (APF; detects mainly hydroxyl radical [^•^OH]) and Singlet Oxygen Sensor Green (SOSG; detects singlet oxygen [^1^O_2_]) were both obtained from Invitrogen (Thermo Fischer Scientific, Waltham, MA USA) and handled according to the manufacturer’s manual. Solutions of PS prepared in phosphate buffer (0.1 M; pH 7.2) in the absence and presence of AU were combined to 3 µM of APF or SOSG in wells of a black flat-bottom 96-well plate (Corning™) and irradiated from above to achieve the desired energy dose. Fluorescence readings were taken immediately after irradiation using the Synergy H1M (Synergy H1 Multi-Mode Reader, BioTek, Winooski, VT, USA). Excitation/emission wavelengths for APF were 490/515 nm, and for SOSG 505/525 nm.

#### Intracellular

A bacterial suspension of ~10^8^ cells/mL of *E*. *faecalis* was prepared in PBS 1×, pelleted by centrifugation (6,500 rpm, 5 minutes) and suspended in 100 µL of a solution of the cell-permeant probe APF (5 µM, in PBS), following by incubation in the dark, at 35 °C, for 30 minutes. Cells were again pelleted, washed to remove the excess of probe and then suspended in 100 µL of PBS (negative control), Ce6 or Ce6 + AU solutions. After 5 minutes of incubation in the dark, suspensions were irradiated with 30 J/cm² (150 mW/cm², 200 s) and then transferred to a black 96-well plate for fluorescence reading (490/515 nm). Control suspensions without APF were used as blanks: cells were incubated in PBS for 30 min, pelleted, incubated with the PS or PS + AU, irradiated ate the same light dose and had their fluorescence determined (490/515 nm). Resultant values were used to normalized APF-treated samples.

### Circular Dichroism spectra and deconvolution

CD spectra were obtained between 190 and 260 nm with a JASCO J-715 CD spectrophotometer (Japan) on nitrogen flush in 1 mm path length quartz cuvettes at room temperature. The peptide concentration was 16 µM, Ce6, CUR and MB concentrations were 84, 68 and 156 µM, respectively. As a positive control of conformational change, a solution containing 5 mM of lysophosphatidylcholine (LPC) was used. CD spectra were typically recorded as an average of ten scans that were obtained in millidegrees and converted to mean-residue ellipticity [θ] (in deg cm^2^ dmol^−1^×10^3^) as follows: spectra were normalized to mean-residue ellipticity (MRE) according to [θ]MRE = θ/(c × l × Nr), where θ is the recorded ellipticity in milli-degrees, c is the peptide concentration in dmol/L, l is the cell path-length in cm, and Nr is the number of residues. The helicity was calculated from the Luo–Baldwin formula Hα = (θ_222nm_ − θ_C_)/(θ^∞^_222nm_ − θ_C_), with θ_C_ = 2200 − 53 T, θ^∞^_222nm_ = (−44,000 + 250 T)(1-k/NResidues), and k = 4 as described for unrestricted peptide^56–58^.

### Zeta potential and particle size

Particle size (mean diameter, nm) and Zeta potential (mV) of *E*. *faecalis* cells treated with AU, Ce6 or Ce6 + AU were measured with a ZetaPALS ZetaPotential Analyzer (Brookhaven Instruments Corporation, Holtsville, NY), equipped with a 677 nm laser and dynamic light-scattering (PCS) at 90° for particle sizing. Zeta potential was determined from electrophoretic mobility μ, at 25 °C, in deionized water. Bacterial suspensions (~10^8^ cells/mL) were incubated with the treatment solutions in the dark for 5 min at a final volume of 3 mL. Suspensions were centrifuged (5000 rpm for 5 min) and pellets were washed twice, being suspended in 2 mL of sterile deionized water. Each value is shown as a mean of at least 3 individual measurements ± mean standard deviation.

### Cell permeabilization assay

Suspensions of *E*. *faecalis* at ~10^8^ cell/mL were incubated with broth (negative control), isopropanol 70% (positive control), AU 16 µM, Ce6 42 or 84 µM, or Ce6 + AU (final volumes were 100 µL) for 5 minutes in the dark. After the incubations, cells were pelleted (6,500 rpm, 5 minutes) and washed twice to remove the treatments and then incubated with propidium iodide (PI, 30 µM in saline) for 5 min. Cells were again pelleted and washed, to be finally suspended in 100 µL of saline for PI fluorescence reading (535/617 nm; Synergy H1 Multi-Mode Reader, BioTek, Winooski, VT, USA).

### Statistical analysis

Data were expressed as the mean plus standard deviation (SD) or medians plus minimum and maximum and were analysed by one-way ANOVA with Tukey’s *post hoc* test, unpaired *t* test or Mann-Whitney test, using GraphPad Prism^®^ Version 5.01 software (GraphPad Software Inc., La Jolla, CA, USA). Differences were considered to be significant when *p* < 0.05 (confidence level of 95%). The maximum acceptable coefficient of variation was set at 25%.

### Data Availability

The datasets generated during and/or analysed during the current study are available from the corresponding author on reasonable request.

## Electronic supplementary material

supplementary figures
