# Supplementary material for: Antimicrobial Photodynamic therapy enhanced by the peptide aurein 1.2
Source: Sci Rep. 2018 Mar 9;8:4212. doi: 10.1038/s41598-018-22687-x (PMC5844988; doi:10.1038/s41598-018-22687-x)
Supplement: Supplementary file 1 — supplementary figures [file 41598_2018_22687_MOESM1_ESM.doc]

**Antimicrobial Photodynamic therapy enhanced by the peptide aurein 1.2**

Laura Marise de Freitas¹; Esteban Nicolás Lorenzón²; Norival Alves Santos-Filho^3^; Lucas Henrique de Paula Zago¹; Marciana Pierina Uliana^4§^; Kleber Thiago de Oliveira^4^; Eduardo Maffud Cilli^3^; Carla Raquel Fontana¹*

Supplementary information

**Figure S1**. **Combined treatment in the dark**. Standardized suspensions of *E. faecalis* were incubated with curcumin (a), chlorin-e6 (b) or methylene blue (c) in combination with aurein 1.2 for 5 minutes in the dark. Columns represent the average of three independent experiments (n=9), and the bars represent the standard deviation.

**Figure S2**. **Photosensitizer uptake in different concentrations**. Standardized suspensions of *Enterococcus faecalis* were incubated with methylene blue (**a**) or chlorin-e6 (**b**) with or without aurein 1.2 (16 µM) for 5 minutes in the dark. The line inside the boxes represent the medians; boxes represent the minimum and maximum values, and whiskers represent the 10-90 percentiles. Four independent assays (n=12). Different letters indicate a significant difference between groups. Kruskal-Wallis test (p<0.0001 for both analyses) with Dunn’s *posthoc*. MB: methylene blue; Ce6: chlorin-e6; CUR: curcumin; AU: aurein 1.2.

**Figure S3. Comparison of susceptibilities to the different treatments among the strains.** Standardized suspensions of the strains were treated with aurein 1.2 (**a**), MB-PDT/MB-PDT+AU (**b**) or Ce6-PDT/Ce6-PDT+AU (**c**). Columns represent the average of at least three independent experiments, and the bars represent the standard deviation

**Figure S4**. **Chemical identification of AU after synthesis**. **a**: Chromatographic profiles of aurein 1.2 crude (black) and pure (red). Retention times are indicated. **b**: mass spectrum of pure aurein 1.2.
